# The Influence of Antibodies against Angiotensin II Type-1 Receptor on the Outcome of Kidney Transplantation: A Single-Center Retrospective Study

**DOI:** 10.3390/jcm12093112

**Published:** 2023-04-25

**Authors:** Vassilis Filiopoulos, Angeliki Vittoraki, Kalliopi Vallianou, Ioannis Bellos, Pavlina Markaki, George Liapis, Smaragdi Marinaki, Aliki Iniotaki, Ioannis N. Boletis

**Affiliations:** 1 Clinic of Nephrology and Renal Transplantation, Medical School of Athens, National and Kapodistrian University, Laiko General Hospital, 11527 Athens, Greece; 2 Immunology Department and National Tissue Typing Center, General Hospital of Athens, ‘Georgios Gennimatas’, 11527 Athens, Greece; 3 1st Department of Pathology, Medical School of Athens, National and Kapodistrian University, Laiko General Hospital, 11527 Athens, Greece

**Keywords:** kidney transplantation, rejection, graft function, anti-angiotensin II type-1 receptor antibodies, non-HLA antibodies

## Abstract

Allo- and autoimmune mechanisms are involved in kidney allograft rejection and loss. This study investigates the impact of anti-angiotensin II type-1 receptor antibodies (anti-AT1RAbs) detected alone or in association with HLA donor-specific antibodies (HLA-DSAs) on the outcome of kidney transplantation (KTx). Anti-AT1RAbs and HLA-DSAs were detected in 71 kidney transplant (KT) recipients who developed biopsy-proven acute or chronic active T-cell rejection (TCMR) (*n* = 51) or antibody-mediated rejection (ABMR) (*n* = 20), forming the rejection group (RG). The control group (CG) included 71 KTx recipients with comparable characteristics without rejection. All patients had been transplanted with negative T/B flow crossmatch (T/BFCXM). The median follow-up period was 3.7 years. Antibodies were determined pre- and periodically post-KTx by Luminex method for HLA-DSAs and enzyme-linked immunosorbent assay for anti-AT1RAbs. Before KTx, twenty-three (32.4%) patients in the RG, sixteen with TCMR and seven with ABMR, were found anti-AT1Rabs-positive (≥10 U/mL) versus eleven (15.5%) patients in the CG (*p* = 0.031). Simultaneous detection of preformed anti-AT1RAbs and HLA-DSAs was found in five patients of the RG versus two of the CG (*p* = 0.355). At the time of transplant biopsy, fifteen (21.1%) patients, four with ABMR and eleven with TCMR, were positive for anti-AT1RAbs. Anti-AT1RAbs and HLA-DSAs were detected simultaneously in 7/15 (46.7%) cases, three with ABMR and four with TCMR. During the follow-up, thirteen (18.3%) patients in the RG, eight with ABMR and five with TCMR, lost their graft compared to one patient (1.4%) in the CG (*p* = 0.001). Six out of thirteen (46.2%) RG patients who lost the graft were found positive for anti-AT1RAbs pretransplant. Patient survival with functioning graft did not differ significantly between anti-AT1Rabs-positive and negative KT recipients (log-rank *p* = 0.88). Simultaneous detection of anti-ATR1Abs and HLA-DSAs did not have a significant influence on patient survival with functioning graft (log-rank *p* = 0.96). Graft function at the end of the follow-up was better, but not significantly, in anti-AT1Rabs-negative patients, with serum creatinine 1.48 [1.20–1.98] mg/dL and eGFR (CKD-EPI) 48.5 [33.5–59.0] mL/min/1.73 m^2^, compared to anti-AT1Rabs-positive ones who had serum creatinine 1.65 [1.24–2.02] mg/dL (*p* = 0.394) and eGFR (CKD-EPI) 47.0 [34.8–60.3] mL/min/1.73 m^2^ (*p* = 0.966). Anti-AT1RAbs detection pretransplant characterizes KT recipients at increased risk of cellular or antibody-mediated rejection. Furthermore, anti-AT1RAbs, detected alone or simultaneously with HLA-DSAs, appear to be associated with impaired graft function, but their role in graft survival has not been documented in this study. Screening for these antibodies appears to complement pretransplant immunological risk assessment.

## 1. Introduction

While the importance of human leukocyte antigen (HLA) immunity in kidney transplantation (KTx) has been well established; the contribution of antibodies against non-HLA antigens in the pathogenesis of allograft rejection is still a subject of ongoing research [1,2]. Furthermore, a synergistic effect between alloimmunity and autoimmunity to self-antigens appear to be associated with adverse outcomes in KTx [3].

Non-HLA antibodies are classified into two main categories: alloantibodies directed against polymorphic antigens that differ between recipient and donor and autoantibodies that recognize self-antigens. Their antigenic targets described thus far include various minor histocompatibility antigens, vascular receptors, adhesion molecules and intermediate filaments [2]. Among non-HLA autoantibodies, anti-angiotensin II type-1 receptor antibodies (anti-AT1RAbs) are the most extensively studied in kidney transplant (KT) recipients [4].

Anti-AT1R-Abs have been detected in the general population and in extreme age groups ranging from infants born from complicated pregnancies to elderly patients with less clear clinical significance. Clinical associations have been reported in preeclampsia, malignant hypertension, Huntington disease, systemic sclerosis and autoimmune diseases associated with negative outcome [5].

The human gene for AT1R is located on chromosome 3 and contains four exons, which differ greatly in transcription and translation rates, thus resulting in several polymorphisms in AT1R [6]. Anti-AT1RAbs that belong to complement fixing IgG1 and IgG3 subclasses recognize epitopes in the second extracellular loop of the angiotensin II type 1 receptor (AT1R), a member of the G protein-coupled receptor family that mediates the majority of physiologic and pathophysiologic actions of angiotensin II. Overactivity of the angiotensin II–AT1R axis leads to hypertension, cardiac hypertrophy and renal fibrosis resulting in substantial cardiovascular morbidity and mortality. Interestingly, AT1R activation by its natural ligand, angiotensin II, is transient, whereas the anti-AT1RAbs binding results in a more sustained and prolonged activation [4,6].

The precise mechanisms of anti-AT1RAbs-mediated graft injury are complex and involve phosphorylation of extracellular signal-regulated kinase 1/2 and activation of transcription factors, activator protein-1 and nuclear factor-kB, in the endothelium and smooth muscle vascular cells promoting proinflammatory, profibrotic and procoagulant processes [7]. AT1R is also expressed on the surface of immune cells, and their stimulation could trigger an immune response that contributes to the inflammatory vascular process. This inflammatory milieu may further increase the endothelial AT1R expression, thus maintaining the vicious cycle of inflammation and subsequent graft injury [7].

Experimental and clinical evidence demonstrate that anti-AT1RAbs both preformed before transplantation and de novo-developed post transplantation characterize patients at increased risk of acute and chronic allograft rejection and also exert a negative impact on long-term allograft outcome [7,8,9]. It has been described that anti-AT1RAbs may contribute to a wide range of kidney abnormalities. Apart from the autoimmune conditions, they have been associated with refractory vascular allograft rejection in the absence of anti-HLA antibodies [10], while they seem to play a role in podocyte injury in association with focal segmental glomerulosclerosis [11]. However, other literature data did not confirm an association between anti-AT1RAbs and kidney transplant outcomes [12]. Furthermore, a synergistic effect between anti-AT1RAbs and HLA-DSAs is difficult to establish [7]. For these reasons it is believed that the clinical relevance of anti-AT1RAbs in KTx deserves further investigation.

The aim of the present single-center study was to evaluate the effect of anti-AT1RAbs that are detected pre- and posttransplant, alone or in association with donor-specific HLA antibodies (HLA-DSAs), on the outcome of KTx. The results of this study are discussed in relation to the observations during the last 17 years since the seminal publication by Dragun et al. [10].

## 2. Materials and Methods

### 2.1. Study Population

**Figure 1:**
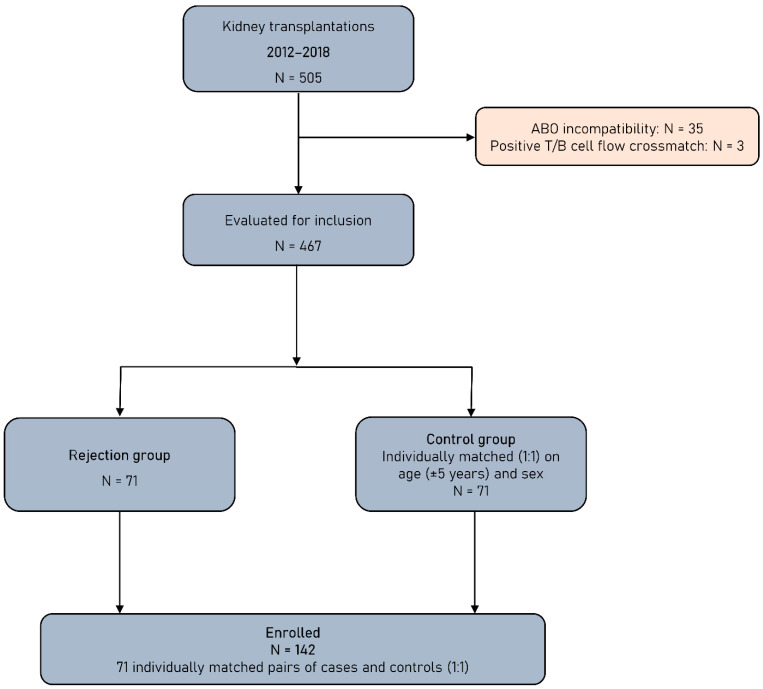
Study flowchart.

This retrospective study includes patients with end-stage renal disease (ESRD) who underwent a KTx from living or deceased donor during seven consecutive years, from 2012 to 2018, in the Kidney Transplant Unit of the General Hospital of Athens “Laiko”. After excluding ABO incompatible transplants and these performed with positive T-cell or B-cell flow cytometry crossmatch (FCXM), all KT recipients of the abovementioned period who experienced acute or chronic rejection were included in this study and formed the rejection group (RG). Patients with biopsy diagnosis other than rejection were not included in this group. Rejection was biopsy-proven and classified according to the Banff 2009–2017 criteria [13,14,15]. Allograft biopsies were performed after clinical indication. Rejection episodes were classified in T-cell-mediated rejection (TCMR) and antibody-mediated rejection (ABMR). Borderline changes were classified as TCMR. KT recipients of the same period with similar baseline characteristics who did not develop a rejection episode were used as the control group (CG). The flowchart of this study is presented in Figure 1.

The entire cohort was followed up from the time of KTx until death, graft loss or 31 December 2020. The study protocol was consistent with the Declaration of Helsinki and was approved by the Institutional Review Board (General Hospital of Athens “Laiko”, protocol code 108/1 February 2018).

For all patients the following parameters were recorded and subsequently analyzed: recipient age, sex, primary renal disease, time on dialysis; donor age, sex and type (living or deceased); expanded criteria donors (ECDs) according to Organ Procurement and Transplantation Network (OPTN) 2002 definition [16]; cold ischemia time; re-transplantation; HLA (A, B, DR) mismatches; hypersensitized recipients defined when panel reactive antibody (PRA) value was above 70%; delayed graft function; induction and maintenance immunosuppression; time from KTx to rejection, time of the follow-up after rejection; follow-up period and therapy with angiotensin-converting enzyme inhibitor (ACEi) or angiotensin receptor blocker (ARB) before and after KTx. The evaluation of graft function included serum creatinine and eGFR by the Chronic Kidney Disease Epidemiology Collaboration (CKD-EPI) equation (2009) and was performed at the time of transplant biopsy, at the end of 1st month post KTx, every 6 months in the follow-up period and at the end of the follow-up.

### 2.2. Immunosuppression

Induction immunosuppression for KTx included therapy with antithymocyte globulin (ATG) or an anti-CD25 monoclonal antibody (basiliximab). The immunosuppressive maintenance regimen consisted of a calcineurin inhibitor (tacrolimus or cyclosporine A), mycophenolate acid (mycophenolate mofetil or mycophenolate sodium) and steroids. Cyclosporine A 2-h target levels were 700–900 mg/dL for the first-year post-transplant and 500–600 mg/dL afterwards, while target tacrolimus trough levels were 8–10 ng/dL and 6–8 ng/dL, respectively. A pulse of 500 mg intravenous methylprednisolone was administered during surgery, followed by 20–40 mg daily for the first two weeks and gradual tapering to the maintenance dose of 4 mg/day after three months. In case pretransplant DSAs at high titers are present and at the discretion of attending physician, plasma exchanges before and/or after KTx (±intravenous immunoglobulin) and/or a single dose of anti-CD20 monoclonal antibody rituximab may be offered and immunosuppression may be occasionally intensified.

### 2.3. Serum Collection and Laboratory Evaluations

All KTx recipients had available pre- and post-KTx sera as well as serum at the time of biopsy, stored at −70 °C until testing. Pre- and post-KTx sera were routinely tested for HLA-DSAs and retrospectively for anti-AT1RAbs. For patients in the RG, these measurements were performed at transplant biopsy as well. Follow-up measurement for HLA-DSAs was performed routinely every year or more often if it was clinically indicated while anti-AT1RAbs were tested once post-KTx, approximately 6 months apart, only if pre-KTx and/or biopsy measurement were positive.

Patients and donors were typed for HLA-A, B, C, DR and DQ antigens using serological (Complement Dependent Cytotoxicity-CDC method) and molecular typing technologies, including sequence-specific oligonucleotide (PCR-SSO) and sequence-specific primer (PCR-SSP) methods following the recommendations of the manufacturer. In cases where HLA-DP-specific antibodies were detected, HLA-DPB1 typing was also performed. The calculated %PRA values and the specificity of IgG anti-HLA class I and II antibodies were determined by LabScreen Single Antigen beads analysis (One Lambda, Canoga Park, CA, USA). For HLA-DSA definition a mean fluorescence intensity (MFI) > 1000 was considered positive. The pre-KTx XM consisted of anti-human globulin (AHG) CDC and T/B flow cytometry XM using current pretransplant serum of the patient. Sera were tested for IgG specificities of HLA class I and II on a Luminex 100 flow multicolor cytometer according to manufacturer’s instructions (One Lambda, Canoga Park, CA, USA). EDTA pretreatment of sera was performed in order to prevent the prozone effect as previously described [17].

Anti-AT1RAbs were determined in patients’ serum using a quantitative solid-phase assay, a commercial enzyme-linked immunosorbent assay (ELISA) (One Lambda Inc.) according to the manufacturer instructions. The detection threshold for anti-AT1RAbs is 2.5 U/mL. Anti-AT1RAbs values ≥ 10 U/mL were considered positive.

### 2.4. Statistical Analysis

Statistical analysis was performed in R-4.0.5 (main packages “survival” [18] and “survminer” [19]). Statistical significance was defined by a two-sided *p*-value threshold of 0.05. The normality of continuous variables was tested by the visual inspection of histograms, along with the statistical significance of the Kolmogorov–Smirnov method. Variables that were deemed to be normally distributed were described by their mean and standard deviation, otherwise the median and interquartile range (IQR) were used. For the comparison of continuous variables between two groups, the Student’s *t*-test or the non-parametric Mann–Whitney U-test were applied. In case of 3 or more groups, the Kruskal–Wallis test was implemented. The comparison of categorical variables was performed with the chi-squared test, while the Fischer’s exact test was applied in case the assumptions of the former were not met. For the endpoint of rejection risk, logistic regression analysis was implemented to provide estimates of odds ratio (OR) and 95% confidence intervals (CI). Both univariable and multivariable models were fitted, adjusting for age, sex, percentage of deceased and extended criteria donors, percentage of hypersensitized recipients and ACEi/ARB therapy and HLA-DSA positivity. Subgroup analysis was conducted based on the aforementioned parameters. On the other hand, the composite outcome of death or graft loss was assessed by Cox proportional hazards regression analysis. The plausibility of the proportional hazards assumption was tested graphically and by using the Schoenfeld’s global test [20]. Kaplan–Meier curves were also constructed and compared with the log-rank test.

## 3. Results

### 3.1. Patients and Transplant Characteristics

As shown in Table 1, demographic and baseline characteristics were similar between the rejection (RG) and control (CG) groups with the only difference being the younger age of KT recipients in the RG (*p* = 0.005).

Out of 142 KT recipients included in this study, 71 developed biopsy-proven acute (*n* = 59) or chronic active (*n* = 12) rejection episodes, which were classified as TCMR subgroup (*n* = 51) or ABMR subgroup (*n* = 20), while in 71 patients no rejection episode was diagnosed. Borderline changes were included in TCMR subgroup. The rejection characteristics are presented in Table 2.

### 3.2. Pretransplant Anti-AT1RAbs and HLA-DSAs

Prior to KTx, 23 (32.4%) KT recipients in the RG were found anti-AT1Rabs-positive compared to 11 (15.5%) KT recipients in the CG (*p* = 0.031) (Table 2). Moreover, anti-AT1RAbs median levels pretransplant were significantly higher in the RG (9.0 U/mL, IQR: 7.4–11.3) compared to the CG (7.6 U/mL, IQR: 5.9–8.7) (*p* < 0.001) (Table 2). Positive anti-AT1RAbs were detected in sixteen of fifty-one (31.3%) KT recipients who developed TCMR and seven of twenty (35%) who developed ABMR (*p* = 0.991). Accordingly, pretransplant anti-AT1RAbs levels were significantly higher in KTx with TCMR (*p* < 0.001) or ABMR (*p* = 0.030) compared to the CG, while no significant difference was detected in pretransplant anti-AT1RAbs median levels between TCMR and ABMR subgroups (*p* = 0.687). The incidence of ABMR and TCMR, when assessed separately, did not differ significantly between anti-AT1RAbs-positive and anti-AT1RAbs-negative KT recipients (*p* = 0.258 and *p* = 0.178, respectively). Likewise, no significant differences were found in the incidence of more severe acute TCMR (Grade IIA and IIB) between AT1RAbs-positive and negative patients.

**Figure 2:**
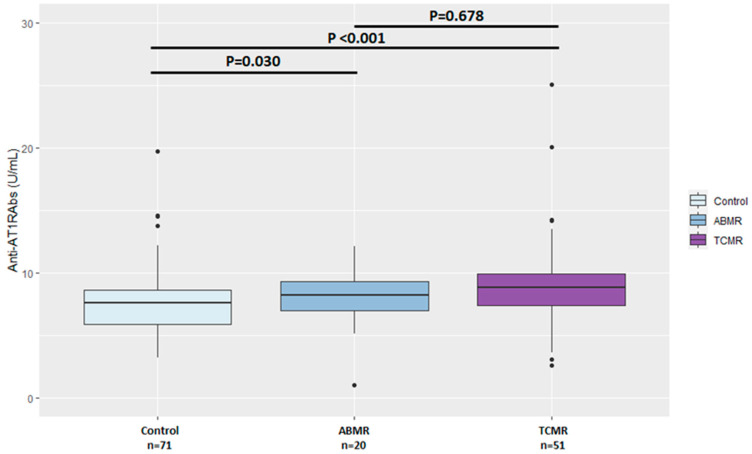
Comparison of pretransplant anti-AT1RAbs levels among the control, T-cell-mediated and antibody-mediated rejection groups. ABMR—antibody-mediated rejection, TCMR—T-cell-mediated rejection.

Comparison of pretransplant anti-AT1RAbs levels among the control, TCMR and ABMR subgroups is shown in Figure 2.

Preformed before KTx HLA-DSAs, not detected with T and B FCXM, were found in nineteen (26.7%) patients in the RG compared to six (8.4%) patients in the CG (*p* = 0.010) (Table 2).

Simultaneous detection of anti-AT1RAbs and HLA-DSAs (double-positive patients) before KTx was found in five patients of the RG, one developed ABMR and four TCMR, versus two patients of the CG (*p* = 0.355). Notably, except for one patient with TCMR, these double-positive patients maintained a functioning graft at the end of the follow-up period. Pretransplant antibody status in the rejection group is shown in Table 3.

### 3.3. Anti-AT1RAbs and HLA-DSAs at the Time of Transplant Biopsy

At the time of transplant biopsy, positive anti-AT1RAbs were identified in 15 (21.1%) patients, with ABMR (*n* = 4) or TCMR (*n* = 11) with median levels 6.9 [4.6–9.5] U/mL. Three out of fifteen patients had de novo-developed anti-AT1RAbs post KTx. Notably, three out of four patients with ABMR and positive anti-AT1RAbs were C4d-negative at transplant biopsy.

Accordingly, HLA-DSAs were identified in the serum of 29 (40.8%) patients with rejection. Twelve out of twenty-nine (41.4%) patients had developed de novo HLA-DSAs post KTx compared to 3/15 (20%) patients with de novo anti-AT1RAbs (*p* = 0.038, chi square test, Yate’s corrected).

Seven out of twenty-nine patients with HLA-DSAs at the time of transplant biopsy had also detectable anti-AT1Rabs. Three of these patients experienced ABMR and four TCMR. In 2/3 ABMR cases, de novo HLA-DSA were present. Notably, four out of seven double-positive patients at transplant biopsy maintained a functioning graft at the end of the follow-up period. Antibodies status at transplant biopsy in patients with TCMR and ABMR, both C4d-negative and C4d-positive, is shown in Table 3.

### 3.4. Anti-AT1RAbs and HLA-DSAs at Follow-Up

After transplant biopsy, follow-up measurements in the RG identified nine patients with positive anti-AT1RAbs out of twenty-six patients with positive anti-AT1RAbs pretransplant and/or at transplant biopsy and 15/71 (21.1%) patients with HLA-DSA, either preformed before KTx or de novo-developed. Of note, two patients developed de novo HLA-DSAs at the follow-up measurement after transplant biopsy. Simultaneous detection of anti-AT1RAbs and HLA-DSAs at the follow-up after transplant biopsy was found in only one patient.

In the CG posttransplant, anti-AT1RAbs remained positive in six out of eleven patients with positive anti-AT1RAbs pretransplant, whereas HLA-DSAs were detected in nine patients, in three of them developed de novo.

Notably, of those patients in both groups that developed de novo HLA-DSAs at the follow-up, none had shown positive anti-AT1RAbs pretransplant.

### 3.5. Graft and Patient Outcomes

First-year and end-of-follow-up serum creatinine and eGFR (CKD-EPI) were significantly better in the CG compared to the RG (Table 2). During the follow-up, thirteen (18.3%) patients in the RG, eight with ABMR and five with TCMR, lost their graft compared to 1/71(1.4%) patient in the CG (*p* = 0.001) (Table 2). Six out of thirteen (46.2%) RG patients who lost the graft were positive for circulating anti-AT1RAbs pretransplant. Accordingly, 4/13 (30.8%) RG patients who lost the graft were positive for anti-HLA-DSA at any time point of antibody detection, with three of them found positive for anti-AT1RAbs as well.

Death occurred in seven (9.9%) patients in the RG and in two (2.8%) in the CG (Table 2) due to common causes for KT recipients (cardiovascular disease, malignancy) irrespective of an immunologic event.

### 3.6. Anti-AT1RAbs Positivity and Transplant Outcomes

The association of pretransplant anti-AT1RAbs positivity with rejection risk was evaluated by logistic regression analysis (Table 4). In crude model, a significant association between pretransplant anti-AT1RAbs positivity with rejection risk was found (OR: 2.61, 95% CI: 1.18–6.08), but this association disappeared after adjustment for age, sex, percentage of deceased and ECDs, percentage of hypersensitized recipients and ACEi/ARB therapy and HLA-DSA positivity.

Furthermore, the influence of pretransplant anti-AT1RAbs positivity on the composite outcome of death or graft loss was assessed by Cox proportional hazards regression analysis (Table 5). Both in crude model and after adjustment for the same variables, no significant association was detected.

**Figure 3:**
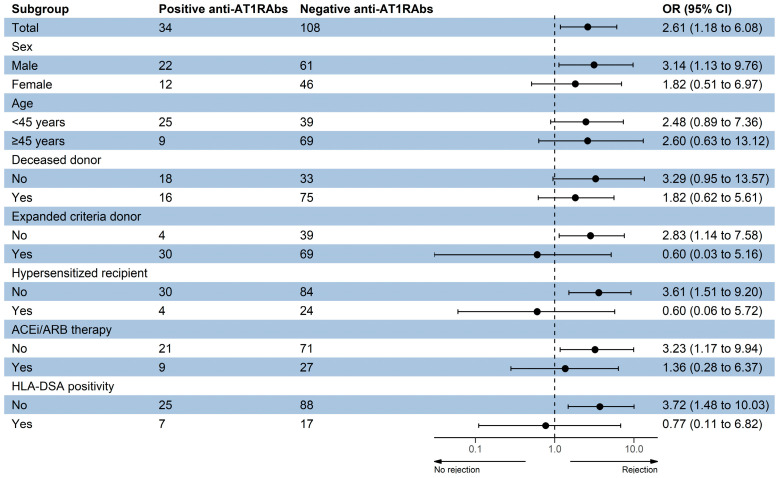
Subgroup analysis of the anti-AT1RAbs positivity effects on rejection risk. ACEi—angiotensin-converting enzyme inhibitor, ARB—angiotensin receptor blocker, HLA—human leukocyte antigen, DSA—donor-specific antibody.

Subgroup analysis of the anti-AT1RAbs positivity effects on rejection risk was conducted by logistic regression analysis based on the aforementioned parameters (Figure 3). Pretransplant anti-AT1RAbs positivity was found to be associated with increased risk of rejection in males, in standard criteria donors, in non-hypersensitized KT recipients, in those not on ACEi/ARB therapy pretransplant and in the absence of preformed HLA-DSAs.

**Figure 4:**
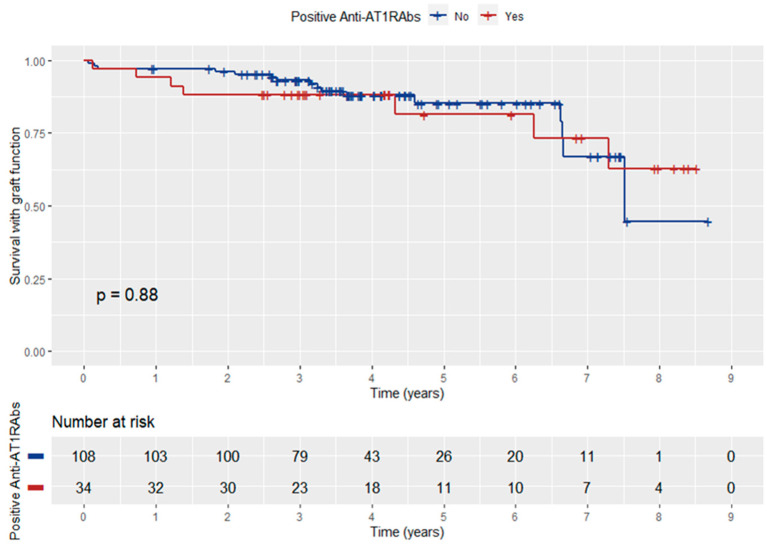
Kaplan–Meier curve depicting the influence of anti-ATR1Abs positivity on patient survival with graft function.

**Figure 5:**
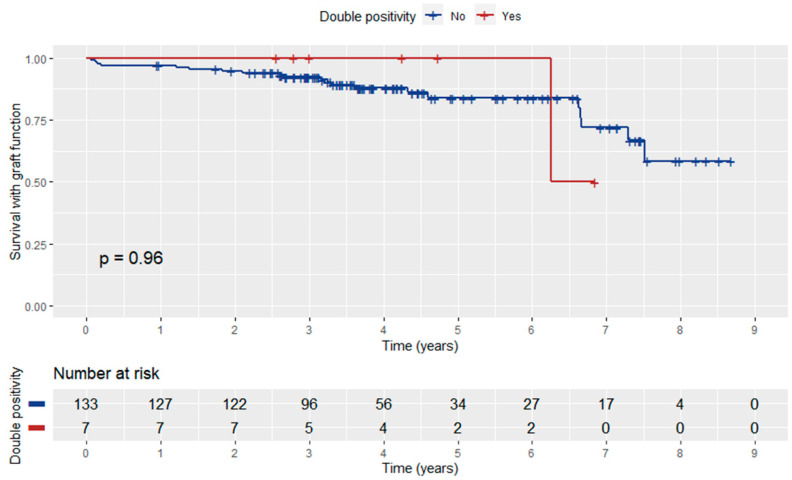
Kaplan–Meier curve depicting the influence of anti-ATR1Abs and HLA-DSA double positivity on patient survival with graft function.

Patient survival with functioning graft did not differ significantly between anti-AT1RAbs-positive and negative KT recipients (log-rank *p* = 0.88) (Figure 4). In addition, anti-ATR1Abs and HLA-DSAs double positivity did not have a significant influence on patient survival with graft function (log-rank *p* = 0.96) (Figure 5).

### 3.7. Graft Function according to Anti-AT1RAbs Status

Graft function at the end of the follow-up was better, but not significantly, in anti-AT1RAbs-negative patients, with serum creatinine 1.48 [1.20–1.98] mg/dL and eGFR (CKD-EPI) 48.5 [33.5–59.0] mL/min/1.73 m^2^, compared to anti-AT1RAbs-positive ones who had serum creatinine 1.65 [1.24–2.02] mg/dL (*p* = 0.394) and eGFR (CKD-EPI) 47.0 [34.8–60.3] mL/min/1.73 m^2^ (*p* = 0.966).

## 4. Discussion

Although the presence of non-HLA immunity before and after clinical kidney transplantation is evident, the contribution to acute or chronic graft damage has been a subject of research for at least 15 years. Both alloimmune and autoimmune mechanisms are understood to be involved in graft rejection, with the latter mainly associated with chronic damage [3,4]. Data with respect to anti-AT1Rabs-mediated graft injury have been discordant. Several studies showed association of anti-AT1RAbs with rejection [8,9,10,21,22,23,24], whereas others, quite numerous, did not show such a correlation [12,25,26,27,28,29,30,31] These conflicting results may be influenced by diverse study populations, various cut-off values for anti-AT1RAbs positivity, different Banff criteria used for rejection (1997–2017), the timing when the indication or protocol biopsy was performed and immunosuppressive treatment [7]. In this retrospective study, we discuss whether the detection of anti-AT1R autoantibodies before KTx, at the time of biopsy-proven episodes of acute or chronic rejection and during the follow-up, can provide information on the immunological risk of transplantation.

The reported prevalence of anti-AT1RAbs is widely variable in KT recipients related not only to different study populations but also to assay variability and lack of a standardized threshold for positivity [7]. In any case, the distribution of a pretransplant sensitization against AT1R above 10 U/mL in all our patients was 23.9% and close to those of several previous studies [7,9]. The cut-off value of 10 U/mL was used in our study following the manufacturer’s recommendation and in concordance with several previous published studies [8,12,22,23,25,27,32] reporting that this is a threshold with good performance in predicting KTx outcomes. However, higher thresholds for positivity have been suggested in other studies [9,21,24,30,31,33,34,35]. The most prevalent cut-off value reported was that of 17 U/mL. Thus, there is currently no uniform standard for the threshold of positivity, and further studies are necessary to validate different cut-offs.

Pretransplant anti-AT1RAbs have been reported to be more prevalent in younger KT recipients [7,28,29]. This is confirmed in the present study as well. An explanation could be that younger patients are more susceptible to ischemic injury secondary to surgical and hemodynamic challenges related to their size as well as to post-KTx infections resulting in the formation of non-HLA antibodies.

In the literature, antibodies pre-formed before ΚTx are often associated with a specific type of rejection. Pretransplant sensitization against AT1R has been associated with both ABMR and TCMR development. According to our results, patients in the RG had significantly higher incidence of pretransplant anti-AT1RAbs compared to the CG in contrast to other observations [9]. These antibodies characterize KT recipients at increased risk of acute or chronic active, cellular or antibody-mediated rejection within 12–375 days post KTx. However, independent association between anti-AT1RAbs positivity and rejection risk has not been documented, although median anti-AT1RAbs levels were higher, non-significantly though, in TCMR patients.

In line with our findings, Lee et al. [35] showed in a multicenter study that a titer of anti-AT1RAbs >9.05 U/mL was significantly associated with three times higher risk of biopsy-proven rejection within a year post transplantation. Consistent with our study, no significant differences were found between Banff scores and rejection severity in the anti-AT1RAbs-positive and anti-AT1Rabs-negative groups [35]. Pretransplant anti-AT1RAbs ≥ 17 U/mL have been significantly associated with a higher incidence of TCMR development in the first-year post-KTx, an observation that cannot be clearly supported by our results [24,36].

There are studies [8,21,33,34] including a recent meta-analysis [37] that found association between high pretransplant anti-AT1RAbs levels (mostly ≥ 17 U/mL) with ABMR in recipients with or without HLA-DSAs. Furthermore, anti-AT1RAbs identification at transplant biopsy, either alone or in combination with HLA-DSAs, indicates the potential involvement of these antibodies in rejection mechanisms and graft outcome. It has been reported that 2–10% of patients with acute rejection are negative for HLA-DSAs and non-HLA antibodies seem to be implicated in rejection mechanisms [26].

The observation in our study that median anti-AT1RAbs levels at the time of rejection (6.9 U/mL) were reduced compared to pre-transplant (9.0 U/mL) confirms previous observations. In the study by Yu et al. [23], median anti-AT1RAbs levels at the time of rejection were 7.9 U/mL and were significantly lower compared to 11.2 U/mL pre-transplantation. The same observation is commented in other studies as well [8,25] and is likely due to the immunosuppression administered and perhaps the absorption of the antibodies into the graft [3].

A detrimental effect of anti-AT1RAbs on kidney graft function emerges, but their role in graft survival has not been documented and deserves a further study and continuous patient monitoring. However, the simultaneous presence of anti-ATR1Abs and HLA-DSAs in patients’ serum was not shown to have a significant influence on patient survival with functioning graft in the present study. The relationship between anti-AT1RAbs and HLA-DSAs remains controversial. Consistent with our findings, several studies reported no association between anti-AT1RAbs and HLA-DSAs in adult [8,37] and pediatric [28] recipients. In contrast to our findings, several studies have shown that recipients with both HLA-DSAs and anti-AT1RAbs had lower graft survival rates and more severe clinical phenotype compared to recipients with either one, suggesting that there is a detrimental synergistic effect of these antibodies on graft survival inducing microvascular inflammation in the allograft [9,22,26,27,32].

It has been reported that patients with anti-AT1RAbs-associated rejection have decreased, or negative C4d deposition in contrast to those with HLA-DSA antibody mediated rejection [10,21,27,32]. In our study, three out of four patients with ABMR and positive anti-AT1RAbs were C4d-negative. However, several studies reported C4d-positive cases of ABMR in association with anti-AT1RAbs [7,22,37]. There are several mechanisms of antibody-mediated graft injury. Apart from strong complement fixing antibodies, B cells produce high-affinity antigen-specific weak or non-complement fixing antibodies. Complement activation as well as mechanisms induced by non-complement fixing antibodies may contribute to endothelial cell damage and subsequently result in allograft injury [38].

In the present study, 12/71 (16.9%) recipients in the rejection group and 6/71 (8.5%) in the control group remained anti-AT1Rabs-positive post-KTx despite the immunosuppression. Furthermore, it is interesting that under immunosuppression at the time of transplant biopsy only 6.25% had developed de novo anti-AT1RAbs, in contrast to 23.07% who developed de novo HLA-DSA post KTx.

Previous studies have reported that the de novo development of HLA-DSAs is significantly higher in recipients with positive anti-AT1RAbs pretransplant [9,24,27,31]. In our study, of those patients in both groups that developed de novo HLA-DSAs at the follow-up, none had shown positive anti-AT1RAbs pretransplant. In agreement with our study, the study by Yu et al. [23] showed that the development of de novo HLA-DSAs does not require pre-existing anti-AT1RAbs.

Pretransplant sensitization against AT1R has been demonstrated as an independent risk factor for graft loss post transplantation [8]. Anti-AT1RAbs can directly activate the endothelium and mediate vascular inflammation, leading to a slower and more chronic injurious process, a progressive decline in renal function and, eventually, allograft loss even in the absence of rejection [27,39].

However, independent association between anti-AT1RAbs positivity and rejection risk or graft loss has not been documented in our study. Furthermore, Lee et al. [35] showed that a titer of anti-AT1RAbs > 9.05 U/mL was not associated with graft failure. Moreover, a French multicentric retrospective study failed to demonstrate an association between pretransplant anti-AT1RAbs levels higher than 10 U/mL and acute rejection, graft survival and 1-year graft function [12]. An Asian retrospective study reported that KT recipients with pretransplant anti-AT1RAbs levels above 10 U/mL had significantly lower allograft survival compared with anti-AT1Rabs-negative ones, and this was attributed to the higher incidence of microvascular inflammation in anti-AT1Rabs-positive KT recipients [27]. In a recent study that included low risk KT recipients, anti-AT1RAbs-positive recipients appear to have persistently lower eGFR compared to anti-AT1RAbs-negative ones throughout the follow-up period, a finding that is emerging in our study as well, at least at the end of the follow-up period [23].

RAS blockade pretransplant may influence anti-AT1RAbs production [6,7]. In our study, ACEi/ARBs treatment was equally distributed between the rejection and control groups. Another potential strength of the present study was the homogeneity of the immunosuppressive regimen. The vast majority received basiliximab as induction, and all patients except for one were on maintenance immunosuppression with calcineurin inhibitor and mycophenolate.

We acknowledge that in this type of cohort-based study, there are several limitations to our results. The retrospective design might be inherently biased by various factors. For obvious reasons, most relevant studies to date are retrospective as shown by a recent review [7]. In addition, the follow-up measurement for anti-AT1RAbs was performed only in pretransplant- and/or at-biopsy-positive patients. Although numerous variables were analyzed, we cannot exclude the possibility that some confounding factors, which were not considered in the present study, could have exerted influence. Finally, we realize that our study does not provide mechanistic answers to the question of how high pretransplant anti-AT1RAbs may trigger rejection. However, anti-AT1RAbs may bind to the allograft immediately following transplantation and initiate pathological pro-inflammatory actions on vascular cells, which are well-defined [7,10,37].

Important areas for investigation remain to identify new endothelial targets for non-HLA antibodies as self-antigens and to clarify mechanisms underlying the production of autoantibodies in the setting of organ transplantation [40,41] Elucidating the processes leading to graft damage by activation of autoimmune mechanisms will facilitate the design of effective management strategies. In parallel, with a long-term follow-up and analysis of large amounts of data using artificial intelligence algorithms, we will be able to determine patterns which characterize KT recipients at high risk of developing autoantibody-mediated injury and to plan their appropriate management.

## 5. Conclusions

In this single-center study performed on a cohort of KT recipients in the recent era of solid phase assay technology for anti-HLA screening, an association between pretransplant immunization against anti-AT1RAbs and increased risk of rejection has been established. Additionally, a trend towards inferior graft function at the end of the follow-up period in anti-AT1RAbs-positive recipients was emerging. However, our study did not confirm an association between pretransplant anti-AT1RAbs and graft survival or de novo HLA-DSA development nor a synergistic effect of anti-AT1RAbs with HLA-DSAs on KTx outcomes.

The literature data have drawn very diverse conclusions regarding the effect of pretransplant anti-AT1RAbs on KT outcomes. A long-term follow-up is needed to clarify the effect of anti-AT1RAbs status on graft outcome. Furthermore, routine anti-AT1RAbs screening, monitoring and therapeutic targeting awaits definitive proof of concept. However, supporting the fact that the cumulative risk characteristics of a patient should always be considered, we would recommend screening for anti-AT1RAbs as an additional risk factor that could be added to the KT recipient’s immunological profile.

## Figures and Tables

**Table 1:** Demographics and baseline characteristics of the entire cohort, as well as the comparison between rejection and control group.

Parameter	All Patients (*n* = 142)	Rejection Group (*n* = 71)	Control Group (*n* = 71)	*p*-Value
Age at transplantation	45.8 ± 13.0	42.8 ± 12.9	48.8 ± 12.5	0.005
Male sex	83 (58.5%)	44 (62.0%)	39 (54.9%)	0.496
Donor age	54.8 ± 13.9	54.6 ± 12.6	54.9 ± 15.3	0.890
Donor male sex	56 (39.4%)	26 (36.6%)	30 (42.2%)	0.607
Deceased donor	91 (64.1%)	40 (56.3%)	51 (71.8%)	0.080
Expanded criteria donor	43 (30.3%)	15 (21.1%)	28 (39.4%)	0.131
Cold ischemia time (h)	17 [15–20.6]	17.5 [16–19.5]	17 [14.8–20.8]	0.316
Time on dialysis (years)	6.3 [2.1–9.5]	4.2 [1.8–8.2]	6.9 [2.6–10.0]	0.103
Primary renal disease				
Diabetes mellitus	4 (2.8%)	3 (4.2%)	1 (1.4%)	0.085
Hypertension	8 (5.6%)	1 (1.4%)	7 (9.8%)	
Glomerulonephritis	33 (23.2%)	18 (25.3%)	15 (21.1%)	
Polycystic kidney disease	21 (14.7%)	8 (11.2)	13 (18.3%)	
Obstructive uropathy/Reflux nephropathy/Interstitial nephritis	18 (12.6%)	13 (18.3%)	5 (7.0%)	
Unknown	50 (35.2%)	25 (35.2%)	25 (35.2%)	
Other	8 (5.6%)	3 (4.2%)	5 (7.0%)	
Re-transplantation	17 (12.0%)	12 (16.9%)	5 (7.0%)	0.119
Hypersensitized recipient	29 (20.4%)	18 (25.3%)	11 (15.4%)	0.271
HLA mismatches	3 [2–4]	3 [3, 4]	3 [2–4]	0.679
Delayed graft function	79 (55.6%)	41 (57.7%)	38 (53.5%)	0.734
Induction immunosuppression				
Basiliximab	133 (93.7%)	68 (95.8%)	65 (91.5%)	0.493
Anti-thymocyte globulin	9 (6.3%)	3 (4.2%)	6 (8.5%)	
Maintenance immunosuppression				
MPA/Tacrolimus	138 (97.2%)	68 (95.8%)	70 (98.6%)	0.620
MPA/Ciclosporin	3 (2.1%)	2 (2.8%)	1 (1.4%	
Tacrolimus/Everolimus	1 (0.7%)	1 (1.4%)	0 (0.0%)	
ACEi/ARB pre-KTx	36/128 (28.1%)	14/60 (23.3%)	22/68 (32.4%)	0.350
ACEi/ARB post-KTx	52 (36.6%)	26 (36.6%)	26 (36.6%)	1
Follow-up period (years)	3.7 [2.9–5.2]	3.4 [2.6–4.8]	3.7 [3.3–5.5]	0.059
Time from transplantation to rejection (days)	-	28 [12–375]	-	N/A
Time of follow-up after rejection (years)	-	2.9 [2–3.9]	-	N/A

Mean ± standard deviation; median [interquartile range]. Bold text indicates statistical significance. HLA—human leukocyte antigen, MPA—mycophenolic acid, ACEi—angiotensin-converting enzyme inhibitor, ARB—angiotensin receptor blocker, KTx—kidney transplantation.

**Table 2:** Main study findings of the entire cohort and comparison between rejection and control group.

Parameter	All Patients (*n* = 142)	Rejection Group (*n* = 71)	Control Group (*n* = 71)	*p*-Value
Rejection classification (Banff 2009–2013)				
ABMR	20 (14.1%)	20 (28.2%)	-	
Acute/active		13 (18.3%)	-	
Chronic active		7 (9.8%)	-	
Borderline changes	8 (5.6%)	8 (11.2%)	-	
TCMR	43 (30.3%)	43 (60.6%)	-	
Acute		38 (53.6%)	-	
Grade IA		*16*		
Grade IB		*10*		
Grade IIA		*6*		
Grade IIB		*6*		
Chronic active		5 (7.0%)	-	
Anti-AT1RAbs pre-KTx (U/mL)	8.2 [6.5–9.8]	9.0 [7.4–11.3]	7.6 [5.9–8.7]	**<0.001**
Anti-AT1Rabs pre-KTx ≥ 10 U/mL	34 (23.9%)	23 (32.4%)	11 (15.5%)	**0.031**
HLA-DSA pre-KTx (MFI > 1000)	25 (17.6%)	19 (26.7%)	6 (8.4%)	**0.010**
First-year serum creatinine (mg/dL)	1.6 [1.3–2.2]	1.8 [1.4–2.9]	1.6 [1.2–2.1]	**0.036**
End-of-follow-up serum creatinine (mg/dL)	1.5 [1.2–2.0]	1.8 [1.4–2.6]	1.3 [1.1–1.6]	**<0.001**
First-year eGFR (CKD-EPI) (mL/min/1.73 m^2^)	42 [31–58]	39 [24.3–58]	43 [33.5–57]	0.229
End-of-follow-up eGFR (CKD-EPI) (mL/min/1.73 m^2^)	47.5 [34.3–60]	38 [23–56]	52 [44.5–65]	**<0.001**
Graft loss	14 (9.9%)	13 (18.3%)	1 (1.4%)	**0.001**
Death	9 (6.3%)	7 (9.9%)	2 (2.8%)	0.166

Mean ± standard deviation; median [interquartile range]. Bold text indicates statistical significance. Anti-AT1RAbs—anti-angiotensin II type-1 receptor antibodies, KTx—kidney transplantation, ABMR—antibody-mediated rejection, TCMR—T-cell-mediated rejection, HLA—human leukocyte antigen, DSA—donor-specific antibody, MFI—mean fluorescent intensity, eGFR—estimated glomerular filtration rate, CKD-EPI—Chronic Kidney Disease Epidemiology Collaboration.

**Table 3:** Antibodies status pretransplant and at biopsy in the rejection group (RG) (*n* = 71).

Antibodies	Pre-KTx	At Biopsy	ABMR C4d (+)	ABMR C4d (−)	TCMR
Anti-AT1RAbs (+) HLA-DSA (−)	18 (25.4%)	8 (11.2%)	1	0	7
Anti-AT1RAbs (−) HLA-DSA (+)	14 (19.7%)	22 (31%)	5	7	10
Anti-AT1RAbs (+) HLA-DSA (+)	5 (7.0%)	7 (9.9%)	0	3	4
Anti-AT1RAbs (−) HLA-DSA (−)	34 (47.9)	34 (47.9%)	1	3	30

KTx—kidney transplantation, ABMR—antibody-mediated rejection, TCMR—T-cell-mediated rejection, Anti-AT1RAbs—anti-angiotensin II type-1 receptor antibodies, HLA—human leukocyte antigen, DSA—donor-specific antibody.

**Table 4:** Association of pretransplant anti-AT1RAbs positivity (≥10 U/mL) with rejection risk.

Model	Odds Ratio	95% Confidence Intervals	*p*-Value
Crude	2.61	1.18–6.08	**0.021**
Model 1	1.88	0.80–4.57	0.153
Model 2	1.72	0.72–4.21	0.227
Model 3	1.72	0.67–4.51	0.258
Model 4	1.97	0.75–5.38	0.174

Model 1—adjusts for recipients’ age and sex; Model 2—additionally, for the percentage of deceased and extended criteria donors; Model 3—additionally, for the percentage of hypersensitized recipients and ACEi/ARB therapy pretransplant; Model 4—additionally, for HLA-DSA positivity. Bold text indicates statistical significance.

**Table 5:** Influence of pretransplant anti-AT1RAb positivity (≥10 U/mL) on the composite outcome of death or graft loss.

Model	Hazard Ratio	95% Confidence Intervals	*p*-Value
Crude	1.07	0.43–2.69	0.878
Model 1	0.79	0.31–2.02	0.619
Model 2	0.82	0.32–2.14	0.690
Model 3	0.43	0.12–1.36	0.215
Model 4	0.49	0.13–1.89	0.302

Model 1—adjusts for recipients’ age and sex; Model 2—additionally, for the percentage of deceased and extended criteria donors; Model 3—additionally, for the percentage of hypersensitized recipients and ACEi/ARB therapy pretransplant; Model 4—additionally, for HLA-DSA positivity.

## Data Availability

Data are not available publicly due to ethical restrictions.
